# Supplementary material for: DNA barcoding echinoderms from the East Coast of South Africa. The challenge to maintain DNA data connected with taxonomy
Source: PLoS One. 2022 Oct 10;17(10):e0270321. doi: 10.1371/journal.pone.0270321 (PMC9550079; doi:10.1371/journal.pone.0270321)
Supplement: Attachment — Submitted filename: Rebuttal letter.docx [file pone.0270321.s007.docx]

**Rebuttal letter**

PONE-D-22-16400

DNA barcoding echinoderms from the East Coast of South Africa. The challenge to maintain DNA data connected with taxonomy

PLOS ONE

**N.B. All replies to the comments of the reviewers start with *****

**Review from the editor**

1. Please ensure that your manuscript meets PLOS ONE's style requirements, including those for file naming. The PLOS ONE style templates can be found at 
<https://journals.plos.org/plosone/s/file?id=wjVg/PLOSOne_formatting_sample_main_body.pdf> and 
<https://journals.plos.org/plosone/s/file?id=ba62/PLOSOne_formatting_sample_title_authors_affiliations.pdf>

***We read again the PLOS ONE's style requirements and adapted our manuscript accordingly.

2. We note that the grant information you provided in the ‘Funding Information’ and ‘Financial Disclosure’ sections do not match.

***We adapted the ‘Funding Information’ section in order to match the Financial disclosure.

3. PLOS requires an ORCID iD for the corresponding author in Editorial Manager on papers submitted after December 6th, 2016. Please ensure that you have an ORCID iD and that it is validated in Editorial Manager. To do this, go to ‘Update my Information’ (in the upper left-hand corner of the main menu), and click on the Fetch/Validate link next to the ORCID field. This will take you to the ORCID site and allow you to create a new iD or authenticate a pre-existing iD in Editorial Manager. Please see the following video for instructions on linking an ORCID iD to your Editorial Manager account: <https://www.youtube.com/watch?v=_xcclfuvtxQ>

***ORCID ID was linked

4. Please include your full ethics statement in the ‘Methods’ section of your manuscript file. In your statement, please include the full name of the IRB or ethics committee who approved or waived your study, as well as whether or not you obtained informed written or verbal consent. If consent was waived for your study

***We added the ethic statement in the material and methods: “All specimens studied here are invertebrates and were collected legally with written permissions from the regional and national South African authorities. The permit number of the expedition of 2016 is RES 2016-02 and was granted by the Department of Agriculture, Forestry and Fisheries (DAFF) and the Department of Environmental Affairs (DEA) of South Africa to Yves Samyn., please include this information in your statement as well.”

5. We note that Figure 1 in your submission contain [map/satellite] images which may be copyrighted.

***We confirm that we have the permission to publish Figure 1 specifically under the CC BY 4.0 license. Indeed, the map was created using the software QGIS v. 3.22.6, the GEBCO Grid and Natural Earth. We added this information and the citation of all three sources in the caption of the figure and uploaded the terms of use of these tools as "Other"" file in the submission.

6. We note that Figure 3 in your submission contain copyrighted images. All PLOS content is published under the Creative Commons Attribution License (CC BY 4.0).
 
***We present a written permission from the copyright holder (a coauthor of this article) to publish these figures specifically under the CC BY 4.0 license (form uploaded as "Other"" file in the submission).


**Reviewer's** **Comments to the Author**

1. Is the manuscript technically sound, and do the data support the conclusions?

Reviewer #1: Yes

2. Has the statistical analysis been performed appropriately and rigorously?

Reviewer #1: I Don't Know

3. Have the authors made all data underlying the findings in their manuscript fully available?

Reviewer #1: No

***All data supporting the findings related to this manuscript were uploaded a public repository (BOLD). As stated in the original submission, data will be released directly upon acceptance to publish this article in PONE. Based on the additional comments of the reviewer, we added all phylogenetic trees as supporting information (see below).

4. Is the manuscript presented in an intelligible fashion and written in standard English?

Reviewer #1: Yes

5. Review Comments to the Author

Reviewer #1: Authors present quite standard procedures broadly utilized for barcoding diverse groups of animals. They also used four standard methods to delimit species based on acquired sequences, and compared them with classic morphological identifications. In Results and Discussion sections they discussed in details differences between those methods that predicted slightly different number of taxa. However, in my opinion the greatest weakened of the presented paper are illustrations. Authors discuss differences between algorithms, however, they didn't present results from each calculation, so it is impossible to compare them by a reader. These results should be at least attached as a supplementary materials.

***We totally agree with this comment. Illustrations are difficult to make for large data sets, and other large scale DNA barcoding studies already published also lack illustrative figures. Our Fig. 2 (general appreciation of the distances between the new sequences and those preexisting) and Fig. 3 (illustrated cases) are there to compensate. The different partitions obtained using the different algorithms were indeed not presented as raw data (they were included in Table 2 but we agree that this is not easy to consult. Hence, we added the putative species ID in the Table S1, which provides all info about all specimens and which can be easily reused by other researchers. The legend of the table was adapted: “**S1 Table. Specimen information.** Columns ABGD, GMYC, bPTP and RESL provide the putative species identifications for each method”.

Moreover, on the Fig 3 authors wrote that trees were calculated using Neighbour Joining method. There is nothing about this method in M&M section.

***Indeed, we forgot to mention that “Neighbour joining trees based on p-distances were constructed using MEGA v7.0.26 (41) in order to illustrate distance among putative species.” This sentence was added in the Materials and Methods.

On the other hand there are no trees calculated using Bayes as mentioned i M&M. That should be definitely corrected or clarified.

***We thank the reviewer for this observation. The original Materials and Methods section was indeed misleading in this regard. Bayesian inferences were generated in order to use them as an input for 2 of the 4 species delimitation methods (GMYC and bPTP), whereas Neighbour joining trees were generated to visualise the distances among the DNA sequences. In addition to the correction described above (concerning the neighbour joining trees), we also modified one sentence in the M&M as follow: “Phylogenetic trees were reconstructed through Bayesian inference (BI) and were used as an input for both GMYC and bPTP” to avoid any confusion. We also provide all 5 phylogenetic trees as Files S1-5 (one tree for each Class).

In general manuscript is well written, however, level of novelty of this research is limited.

***Thank you. “Level of novelty” is of course a relative appreciation. We agree that we do not provide a lot of firm conclusions, but we present data and analyses that will undoubtedly be useful for the taxonomy and systematics of echinoderms, and for future biodiversity inventories.

6. PLOS authors have the option to publish the peer review history of their article ([what does this mean?](https://eur02.safelinks.protection.outlook.com/?url=https%3A%2F%2Fjournals.plos.org%2Fplosone%2Fs%2Feditorial-and-peer-review-process%23loc-peer-review-history&data=05%7C01%7Cgsonet%40naturalsciences.be%7Ccd2b242d47e848c21e0308da5e97a87f%7C4da8d35db472419c9e15665786aadbfb%7C1%7C0%7C637926303745458496%7CUnknown%7CTWFpbGZsb3d8eyJWIjoiMC4wLjAwMDAiLCJQIjoiV2luMzIiLCJBTiI6Ik1haWwiLCJXVCI6Mn0%3D%7C3000%7C%7C%7C&sdata=DWd3NQxl6w405HrT9IaK4Mz%2B3psJWpM%2FW6wdtJmGEFI%3D&reserved=0)). If published, this will include your full peer review and any attached files.

**Do you want your identity to be public for this peer review?** For information about this choice, including consent withdrawal, please see our [Privacy Policy](https://eur02.safelinks.protection.outlook.com/?url=https%3A%2F%2Fwww.plos.org%2Fprivacy-policy&data=05%7C01%7Cgsonet%40naturalsciences.be%7Ccd2b242d47e848c21e0308da5e97a87f%7C4da8d35db472419c9e15665786aadbfb%7C1%7C0%7C637926303745458496%7CUnknown%7CTWFpbGZsb3d8eyJWIjoiMC4wLjAwMDAiLCJQIjoiV2luMzIiLCJBTiI6Ik1haWwiLCJXVCI6Mn0%3D%7C3000%7C%7C%7C&sdata=vjlwo9cpL8ysAgf5jdYfglU41kkFBGZVBcf5YmNiXgI%3D&reserved=0).

Reviewer #1: No

While revising your submission, please upload your figure files to the Preflight Analysis and Conversion Engine (PACE) digital diagnostic tool, [https://pacev2.apexcovantage.com/](https://eur02.safelinks.protection.outlook.com/?url=https%3A%2F%2Fpacev2.apexcovantage.com%2F&data=05%7C01%7Cgsonet%40naturalsciences.be%7Ccd2b242d47e848c21e0308da5e97a87f%7C4da8d35db472419c9e15665786aadbfb%7C1%7C0%7C637926303745458496%7CUnknown%7CTWFpbGZsb3d8eyJWIjoiMC4wLjAwMDAiLCJQIjoiV2luMzIiLCJBTiI6Ik1haWwiLCJXVCI6Mn0%3D%7C3000%7C%7C%7C&sdata=dmIt4YRMHTLI75mvSnTyWk6A%2BBJ7d3%2BOGtCoqRPCKow%3D&reserved=0). PACE helps ensure that figures meet PLOS requirements. To use PACE, you must first register as a user. Registration is free. Then, login and navigate to the UPLOAD tab, where you will find detailed instructions on how to use the tool. If you encounter any issues or have any questions when using PACE, please email PLOS at [figures@plos.org](mailto:figures@plos.org). Please note that Supporting Information files do not need this step.

***All three figures passed the PACE quality control.
