# Supplementary material for: DNA barcoding echinoderms from the East Coast of South Africa. The challenge to maintain DNA data connected with taxonomy
Source: PLoS One. 2022 Oct 10;17(10):e0270321. doi: 10.1371/journal.pone.0270321 (PMC9550079; doi:10.1371/journal.pone.0270321)
Supplement: Attachment — Submitted filename: Rebuttal letter rev2.docx [file pone.0270321.s008.docx]

Rebuttal letter

*Nota bene*:	**Responses to the reviewer are in bold and follow the signs “>>>”.**

**Actions taken in the manuscript follow the signs “->”.**

Reviewers' comments:

Reviewer's Responses to Questions

Comments to the Author

Reviewer #2: All comments have been addressed

2. Is the manuscript technically sound, and do the data support the conclusions?

Reviewer #2: Yes

3. Has the statistical analysis been performed appropriately and rigorously?

Reviewer #2: Yes

4. Have the authors made all data underlying the findings in their manuscript fully available?

Reviewer #2: Yes

5. Is the manuscript presented in an intelligible fashion and written in standard English?

Reviewer #2: Yes

6. Review Comments to the Author

Reviewer #2: The authors appear to have addressed all of the matters raised by previous reviews. They have done a good job of analysing and presenting a complex mix of existing and new DNA barcode data in a poorly studied group. It is often very difficult to combine new barcode data with existing data which not infrequently contains sequences attributed to incorrect species.

**>>> Thank you. Yes, we agree that it represented a major part of the effort involved in this manuscript**

The authors point out the limitations in their dataset and highlight areas that need further clarification in future.

**>>> Yes, this is important of course**

I make only minor comments on the methods which the authors should address in the final version of the manuscript, nevertheless I reccomend the manuscript be accepted for publication:

Lines 161-163: NJ trees were based on p-distances. Building trees based on uncorrected distances has not been acceptable practice since model-based distance corrections became commonplace in the 1980s. While Bayesian or ML trees would be vastly preferable, NJ trees are acceptable for barcode analyses if based on corrected distances. MEGA has many options for distance correction. BOLD uses K2P-distances by default so that would be a good choice. Regardless, if the NJ trees are never shown then what is their purpose? I suggest deleting all reference to NJ trees and using the Bayesian trees.

**>>> We totally agree that uncorrected distances are not recommended for phylogenetic tree reconstructions. That is why we used Bayesian inference to reconstruct the ultrametric phylogenetic trees that were used for tree-based species delimitations. Here we simply used uncorrected distances to illustrate the proportion of substitutions observed among some putative species in Fig. 3. In our opinion, these trees are good for illustration (because they are without any *a priori* model-based distances) if they are introduced correctly to avoid risks of misinterpretation. We understand that this is debatable and apply the changes suggested by the reviewer**

- **we remove the reference to the NJ tree and Fig. 3. which are only there to illustrate a content, which is already fully described in the main text and in the tables.**

Lines 163-164: On what date was the search function used on BOLD to retrieve sequences? This information is vital for reproducibility of methods.

**>>> We agree that it is of primary importance and the information was already in reference ‘25’ cited on line 163.**

- **To make it more visible, we repeat it in the main text by adding “(…) on the 24 May 2022”.**

Lines 181-183: Although BOLD produces BINs based on the RESL algorithm, it is not correct to equate BINs to the results of RESL analysis because BOLD uses additional criteria to assign sequences to BINs, including data quality standards (sequence length, etc.). It is common to have only a subset of sequences in a dataset assigned to BINs. However, it is possible to perform RESL analysis on BOLD for all sequences in your dataset, regardless of whether BOLD assigns them to a BIN. Besides which, BINs are not assigned immediately but only periodically as BOLD analyses new sequences for BIN assignment. So, did the authors perform RESL analyses or not? Were all their sequences assigned to BINs? The authors need to clarify exactly what they did.

**>>> We thank the reviewer for these precisions. We did not perform RESL analyses ourselves. We indeed used the BIN IDs when they became available on BOLD. For a few sequences without a BIN, we followed the advice of the support team of BOLD (email of 12 June 2020) who advised to use the “Cluster Sequences tool” in the workbench to group COI sequences into OTUs. If the other sequences in the OTU have a BIN, it is likely that those without a BIN belong to that same one, but may not be included in it for some quality check reasons, otherwise they would have been assigned to that BIN.**

- **In the text, we replaced “RESL” by “BIN”. The way we obtained the BINs was already clarified in lines 182-187 (but the “RESL” abbreviation was inded wrongly used). It reads now: “The BINs were obtained on BOLD. (…). When the sequence overlap with the BOLD sequences was lower than 300 bp and a BIN was not attributed directly by BOLD, the BIN of the best match above 99% similarity was assigned to the record.”**
